# “One for All”: Functional Transfer of OMV-Mediated Polymyxin B Resistance From *Salmonella enterica* sv. Typhi Δ*tolR* and Δ*degS* to Susceptible Bacteria

**DOI:** 10.3389/fmicb.2021.672467

**Published:** 2021-05-05

**Authors:** Pedro Marchant, Alexander Carreño, Eduardo Vivanco, Andrés Silva, Jan Nevermann, Carolina Otero, Eyleen Araya, Fernando Gil, Iván L. Calderón, Juan A. Fuentes

**Affiliations:** ^1^Laboratorio de Genética y Patogénesis Bacteriana, Departamento de Ciencias Biológicas, Facultad de Ciencias de la Vida, Universidad Andres Bello, Santiago, Chile; ^2^Facultad de Ciencias Exactas, Center of Applied NanoSciences (CANS), Universidad Andres Bello, Santiago, Chile; ^3^Escuela de Química y Farmacia, Facultad de Medicina, Universidad Andres Bello, Santiago, Chile; ^4^Departamento de Ciencias Químicas, Facultad de Ciencias Exactas, Universidad Andres Bello, Santiago, Chile; ^5^Microbiota-Host Interactions and Clostridia Research Group, Universidad Andres Bello, Santiago, Chile; ^6^ANID-Millennium Science Initiative Program-Millennium Nucleus in the Biology of the Intestinal Microbiota, Santiago, Chile; ^7^Laboratorio de RNAs Bacterianos, Departamento de Ciencias Biológicas, Facultad de Ciencias de la Vida, Universidad Andres Bello, Santiago, Chile

**Keywords:** *Salmonella* Typhi, outer membrane vesicles, OMVs, polymyxin, *rfaE*, *degS*, *tolR*, antibiotic resistance

## Abstract

The appearance of multi-resistant strains has contributed to reintroducing polymyxin as the last-line therapy. Although polymyxin resistance is based on bacterial envelope changes, other resistance mechanisms are being reported. Outer membrane vesicles (OMVs) are nanosized proteoliposomes secreted from the outer membrane of Gram-negative bacteria. In some bacteria, OMVs have shown to provide resistance to diverse antimicrobial agents either by sequestering and/or expelling the harmful agent from the bacterial envelope. Nevertheless, the participation of OMVs in polymyxin resistance has not yet been explored in *S.* Typhi, and neither OMVs derived from hypervesiculating mutants. In this work, we explored whether OMVs produced by the hypervesiculating strains *Salmonella* Typhi Δ*rfaE* (LPS synthesis), Δ*tolR* (bacterial envelope) and Δ*degS* (misfolded proteins and σ^*E*^ activation) exhibit protective properties against polymyxin B. We found that the OMVs extracted from *S.* Typhi Δ*tolR* and Δ*degS* protect *S.* Typhi WT from polymyxin B in a concentration-depending manner. By contrast, the protective effect exerted by OMVs from *S.* Typhi WT and *S.* Typhi Δ*rfaE* is much lower. This effect is achieved by the sequestration of polymyxin B, as assessed by the more positive Zeta potential of OMVs with polymyxin B and the diminished antibiotic’s availability when coincubated with OMVs. We also found that *S.* Typhi Δ*tolR* exhibited an increased MIC of polymyxin B. Finally, we determined that *S.* Typhi Δ*tolR* and *S.* Typhi Δ*degS*, at a lesser level, can functionally and transiently transfer the OMV-mediated polymyxin B resistance to susceptible bacteria in cocultures. This work shows that mutants in genes related to OMVs biogenesis can release vesicles with improved abilities to protect bacteria against membrane-active agents. Since mutations affecting OMV biogenesis can involve the bacterial envelope, mutants with increased resistance to membrane-acting agents that, in turn, produce protective OMVs with a high vesiculation rate (e.g., *S.* Typhi Δ*tolR*) can arise. Such mutants can functionally transfer the resistance to surrounding bacteria via OMVs, diminishing the effective concentration of the antimicrobial agent and potentially favoring the selection of spontaneous resistant strains in the environment. This phenomenon might be considered the source for the emergence of polymyxin resistance in an entire bacterial community.

## Introduction

*Salmonella enterica* serovar Typhi (*S.* Typhi) is the etiologic agent of typhoid fever in humans, a disease producing hundreds of deaths worldwide per year, especially in developing countries (Ajibola et al., 2018; Bhutta et al., 2018; Johnson et al., 2018). *S.* Typhi infection begins with the ingestion of contaminated water or food (Hook et al., 1990). Bacteria reach the small intestine and promote their internalization through intestinal epithelial cells and the M cells of the Peyer’s patches, reaching the underlying lymphoid tissue. At this point, bacteria are disseminated to deep organs inside dendritic cells, macrophages, or neutrophils (Galan, 1996; Miao et al., 2003), allowing the systemic bacterial spread (typhoid fever). During the infection, host cells from the innate immune defense respond to bacterial elements [e.g., lipopolysaccharides (LPS)], producing cationic antimicrobial peptides (<100 amino acids), which interact with the anionic bacterial membranes to produce microbial death (Hancock and Scott, 2000; Zasloff, 2002).

The full progression of typhoid fever was commonly observed in the pre-antibiotic era. Nevertheless, the emergence of multi-resistant strains represents a severe problem, with an increased recurrence rate of the disease (Als et al., 2018; Schwartz and Morris, 2018). The appearance of multidrug-resistant strains introduced the use of quinolones (Parry et al., 2013; Karkey et al., 2018), albeit the appearance of quinolone-resistance variants led to increased use of azithromycin and third-generation cephalosporins (Pandit et al., 2007; Karkey et al., 2018). Unfortunately, *S.* Typhi strains that produce extended-spectrum β-lactamase have been increasingly reported (Ahamed Riyaaz et al., 2018; Karkey et al., 2018). This scenario underlines the importance of studying antibiotic resistance in *S.* Typhi.

The appearance of multi-resistant strains (Payne et al., 2007; Kumarasamy et al., 2010; Cornaglia et al., 2011) contributed to reintroducing polymyxins (cationic peptides) as the last-line therapy when more commonly used antibiotics are inefficient (Velkov et al., 2013; Garg et al., 2017). It is generally accepted that polymyxins exert their antimicrobial activity by first interacting with the outer-membrane components of Gram-Negative bacteria. Polymyxins are peptides carrying a hydrophobic acyl tail with positively charged residues (Daugelavicius et al., 2000). Due to their cationic and amphipathic nature, polymyxins electrostatically interact with the negatively charged lipopolysaccharides (LPS). Interestingly, evidence shows that a fluorescent polymyxin derivative can also bind to unspecified outer membrane proteins (van der Meijden and Robinson, 2015), strongly suggesting that polymyxins interact with different kinds of molecules in the outer membrane of Gram-negative bacteria. At this point, the “self-promoted uptake” occurs, a process based on the presence of the hydrophobic acyl tail of polymyxin, enabling polymyxin to insert into the outer membrane by displacing membrane-stabilizing cationic ions, such as Ca^2+^ and Mg^2+^, and interacting with the lipid A, a recognized polymyxin-binding target in the outer membrane (Velkov et al., 2010; Trimble et al., 2016). Polymyxin insertion in the outer membrane weakens the packing of contiguous lipid A, disrupting the permeability barrier (Falagas and Kasiakou, 2006; Velkov et al., 2013; Trimble et al., 2016). Although subsequent steps are not fully elucidated, the evidence argues for the fusion of the inner membrane’s outer leaflets with the outer membrane’s inner leaflet to form pores, leading to an osmotic imbalance and a subsequent death (Daugelavicius et al., 2000). At present, an increasing number of reports regarding polymyxin resistance are being published (Li et al., 2019). Some chromosomal mutations have been associated with increased resistance to polymyxins, including modifications of the *pmrCAB* and *phoPQ* operons, among other genes (Li et al., 2019). In *Salmonella enterica* and *Escherichia coli*, many of those mutations lead to LPS modifications, decreasing the anionic charges and diminishing the electrostatic binding of polymyxin to bacterial outer membranes (Li et al., 2019). Nevertheless, the spread of polymyxin resistance has been only associated with the transferable gene *mcr*, which encodes a phosphoethanolamine transferase that modifies the lipid A (Olaitan et al., 2014). Although significant advances in understanding polymyxin-resistance mechanisms have been made, this area needs further research.

OMVs are nanosized proteoliposomes formed and secreted from the outer membrane of Gram-negative bacteria (Kulp and Kuehn, 2010). OMVs biogenesis mainly relies on (1) changes in LPS composition, (2) the dissociation of the outer membrane in specific zones, and (3) accumulation of misfolded proteins in the periplasm (Kulp and Kuehn, 2010; Kulkarni and Jagannadham, 2014). OMVs play different roles in the bacterial life cycle, such as delivering proteins and defense against harmful agents such as phages and antibiotics, among other functions (Kulp and Kuehn, 2010; Jan, 2017). OMVs contribute to resistance to diverse molecules with antimicrobial properties either by sequestering (“decoy”) and/or expelling the harmful agent from the bacterial envelope. Some examples include resistance to toluene in *Pseudomonas putida*, chlorhexidine in *Porphyromonas gingivalis*, and polymyxins in *Escherichia coli* (Grenier et al., 1995; Kobayashi et al., 2000; Manning and Kuehn, 2011; Roszkowiak et al., 2019). In this context, the participation of OMVs in polymyxin resistance has not yet been explored in *S.* Typhi, and neither OMVs derived from hypervesiculating mutants.

A recent study screened 15,000 mutants searching for genes involved in OMVs biogenesis in *S.* Typhi (Nevermann et al., 2019). *S.* Typhi Δ*rfaE*, Δ*tolR*, and Δ*degS* showed some of the most potent hypervesiculation phenotypes compared with the wild type (WT) (Nevermann et al., 2019). In particular, the *rfaE* (*waaE*) gene product is thought to be involved in the formation of ADP-L-glycero-D-manno-heptose of LPS. *Salmonella* Typhimurium Δ*rfaE* mutants synthesize heptose-deficient LPS, exhibiting only lipid A and 3-deoxy-D-manno-octusolonic (KDO) acid (Jin et al., 2001). TolR is an inner membrane protein belonging to the trans-envelope Tol-Pal complex, highly conserved in Gram-negative bacteria (Sturgis, 2001). In *E. coli*, TolR contributes to maintaining the envelope structure and participates in the retrograde phospholipid transport (Muller et al., 1993; Boags et al., 2019). In *E. coli*, DegS is a serine protease harboring a PDZ domain that inhibits the protease activity in the absence of stress (Alba et al., 2001). Under stress, mainly due to overexpression of outer membrane proteins, misfolded proteins accumulate in the periplasm, activate the DegS protease activity to cleave the anti-sigma factor RseA, releasing σ^*E*^. In *Salmonella* Typhimurium, σ^*E*^ is required under envelope stress and in the presence of antimicrobial peptides (Testerman et al., 2002; Palmer and Slauch, 2020).

In this work, we explored the protective effect of OMVs produced by *S.* Typhi WT, Δ*rfaE*, Δ*tolR*, and Δ*degS*. Besides, we tested whether the polymyxin resistance can be functionally transferred to polymyxin-susceptible bacteria. We found that *S.* Typhi OMVs protect bacteria against polymyxin B in a concentration-dependent manner by sequestering the antibiotic, where OMVs from *S.* Typhi Δ*tolR* and Δ*degS* showed the highest protection levels. OMVs from *S.* Typhi Δ*tolR* also protected *Candida albicans* against limonene, a membrane-active antimicrobial agent. Finally, we found that *S.* Typhi Δ*tolR* and, at a lesser level, *S.* Typhi Δ*degS* can functionally transfer the OMV-mediated polymyxin B resistance to susceptible bacteria. This study underlines that some mutations affecting vesiculation in a population can increase polymyxin resistance in a bacterial community.

## Materials and Methods

### Bacterial Strains, Media, and Culture Conditions

*Salmonella* Typhi strain STH2370 (*S.* Typhi WT) was used as parental strain (Valenzuela et al., 2014). *S.* Typhi Δ*rfaE*::FRT, Δ*tolR*::FRT, and Δ*degS*::FRT were previously reported (Nevermann et al., 2019). *S.* Typhimurium LT2 *ompD*::Mu*d*-J (Lac^+^) was kindly provided by Dr. Guido Mora (Santiviago et al., 2003). Strains were routinely grown in liquid culture using Luria Bertani medium (Bacto peptone, 10 g/L; Bacto yeast extract, 5 g/L; NaCl, 5 g/L; prepared in phosphate buffer pH 7.0) at 37 °C with shaking. When required, the medium was supplemented with X-gal (5-bromo-4-chloro-3-indolyl-β-D-galactopyranoside) (40 μg/mL) and/or agar (15 g/L). *Candida albicans* corresponds to a clinical isolate from the Hospital Clínico de la Universidad de Chile (Carreno et al., 2018; Carreño et al., 2021). Yeasts were cultured in Sabouraud agar (Bacto peptone, 10 g/L; glucose, 40 g/L; agar, 15 g/L; pH 5.6) at 28°C.

### OMV Isolation, Quantification, and Size Measurement

To isolate OMVs (Liu et al., 2016b; Nevermann et al., 2019), bacteria were grown in LB at 37 °C with shaking (OD_600_ = 1.1) before being centrifuged 10 min at 5,400 × *g* at 4°C. The pellet was discarded, and the supernatant was filtered (0.45 μm), ultrafiltered (Ultracel^®^ 100 kDa ultrafiltration discs, Amicon^®^ Bioseparations), and ultracentrifuged 3 h at 150,000 × *g* at 4°C. The supernatant was discarded, and the pellet was resuspended in 1 mL DPBS (Dulbecco’s phosphate-buffered saline) (Gibco). OMVs were stored at −20°C until their use. We quantified OMV yield by determining the protein content (BCA assay) and/or the lipid content (FM4-64 molecular probe) (McBroom et al., 2006; Deatherage et al., 2009). We determined OMV size as described (Deatherage et al., 2009; Nevermann et al., 2019). Results were presented as the diameter, classified into the median (P50), and P25 and P75.

### Transmission Electron Microscopy (TEM)

OMV extracts were bound to formvar-coated slot grids, stained with 1% aqueous uranyl acetate for 1 min, and viewed with a Philips Tecnai 12 (Biotwin) transmission electron microscope, as described (Nevermann et al., 2019).

### Determination of Zeta Potential

The Zeta potential of OMVs was measured at room temperature (25°C) by a Zetasizer Nano series MPT-Z multi Purpose Titrator (Malvern, United Kingdom). The device was equipped with a Helium-Neon laser (633 nm) as a light source. The detection angle of Zetasizer at aqueous media was 173.13° (measurement range: 0.3 nM—10 μm diameter). Capillary cells DTS 1070 were used. To measure the Zeta potential, polymyxin B and OMVs were resuspended in Mili-Q water.

### Determination of Minimal Inhibitory Concentration (MIC)

Minimum inhibitory concentration (MIC) was obtained by broth dilution as described (Cuenca-Estrella et al., 2003), with modifications. Briefly, bacteria and yeast were previously cultured as described above. Microorganisms were then diluted in PBS (0.5 McFarland) and then diluted again (1000-fold) in LB for bacteria or Bacto Tryptic Soy broth (Sigma Aldrich) for yeasts, before seeding a 96-well plate. In each well, 180 μL of this dilution was placed, along with 10 μL polymyxin B sulfate (AppliChem GmbH), ciprofloxacin (Sigma Aldrich) or (*R*)-(+)-limonene [(+)-*p*-mentha-1,8-diene,(+)-carvene,(*R*)-4-isopropenyl-1-methyl-1-cyclohexene] (Sigma Aldrich) (stock prepared in 95% ethanol) to achieve a final known concentration, and 10 μL DBPS. When indicated, the 10 μL DBPS were replaced by 10 μL of purified OMVs to achieve a final known concentration. Alternatively, and when indicated, the 10 μL DBPS were replaced by 10 μL of bacterial supernatant. To obtain the supernatant, bacteria were cultured in LB as stated above (OD_600_ = 1.0–1.3), centrifuged 10 min at 5,400 × *g* at 4°C, the pellet was discarded, and the supernatant fraction was filtered (0.45 μm). The 96-well plates were incubated overnight at 37°C (bacteria) or 24 h at 28°C (yeasts). The MIC was determined by OD_600_ measurement and corroborated by visual inspection and plating onto agar plates.

### Estimation of Polymyxin Sequestration by OMVs

To determine Zeta potential changes due to the interaction with polymyxin B, OMV extracts (50 μg/mL) were mixed with 0, 5, 50, or 100 μg/mL polymyxin B and incubated 30 min at 37°C with gentle agitation. The mixture was ultrafiltered in Ultracel^®^ 100 kDa ultrafiltration column (Amicon^®^ Bioseparations) at 5,400 × *g* for 10 min to remove the unbound polymyxin B. The ultrafiltrated obtained with 100 μg/mL polymyxin B was reserved (see below). OMVs were resuspended in 1 volume of Mili-Q water before measuring the Zeta potential. As control of polymyxin B removal, we measured the Zeta potential of water alone (−0.05 ± 0.35 mV), water + 100 μg/mL polymyxin B (5.57 ± 2.98 mV), and water + 100 μg/mL polymyxin ultrafiltered and resuspended in 1 volume of Mili-Q water (0.73 ± 0.43 mV). To estimate the relative amount of polymyxin B sequestered by OMVs, the reserved ultrafiltered was diluted 10 times in LB and then serially diluted in LB to determine the last dilution that inhibited the *S.* Typhi WT growth. As a control, we used a solution with no OMVs.

### Protection Assay of a Reporter Strain (“One for All”)

Approximately 5 × 10^5^ CFU/mL of *S.* Typhi WT or mutant derivatives were mixed with 5 × 10^6^ CFU/mL of *S.* Typhimurium *ompD*::Mu*d*-J. Bacteria were previously washed three times with PBS to remove all the accumulated OMVs and resuspended in LB. This mixture was incubated 0 (with no incubation), 1 or 2 h at 37°C with shaking before adding polymyxin B (final concentration: 2.5 μg/mL). Bacterial mixtures were incubated at 37°C with shaking overnight, and CFUs were counted on LB agar with X-gal (*S.* Typhi strains: white colonies, *S.* Typhimurium reporter strain: blue colonies). Alternatively, the serovar was corroborated by PCR (Fuentes et al., 2008) for some colonies. As a control, the strains were tested separately under this same procedure.

### Determination of μ and t_*d*_

Bacteria were cultured in LB as described above, and OD_600_ was recorded every 10 min to construct a growth curve. To calculate μ and t_*d*_, we used:

$$\mu =\frac{\ln\left(N\right)-\text{ln}\,{\left(N\right)}_{0}}{t-t_{0}}$$

$$t_{d}=\frac{0.693}{\mu }\times 60$$

Where μ (h^–1^): growth rate; *N*: bacteria at the end of the logarithmic phase (OD_600_); *N*_0_ bacteria at the beginning of the logarithmic phase (OD_600_); *t*: time at the end of the logarithmic phase (h); *t*_0_: time at the end of the logarithmic phase (h); t_*d*_: duplication time (min).

### LPS Profile Determination

To observe the LPS profile of OMVs, we followed a protocol previously reported (Kulikov et al., 2019). OMVs were extracted as described above prior to being mixed with 1 volume of lysis buffer (2% w/v of SDS, 4% v/v of 2-mercaptoethanol, 10% v/v glycerol, 1 M Tris-HCl pH 6.8, and 0.05% w/v bromophenol blue). The mixture was incubated at 95 °C for 10 min, cooled to room temperature, and 10 μL of 2.5 mg/mL Proteinase K solution made in the lysis buffer was added before being incubated at 56°C for 1 h in a heating shaker. The preparation obtained was directly loaded on a conventional protein SDS polyacrylamide gel with 12% acrylamide (19:1 acrylamide:bisacrylamide), and it was run at a constant 20 mA current in Tris-glycine-SDS buffer. In order to observe the LPS profile, the gel was treated with fixer-oxidizer solution (40% v/v ethanol, 5% v/v acetic acid, 1% w/v sodium periodate, Milli-Q water up to 1 v) and incubated for 15 min. Then, the gel was washed three times with distilled water (7 min each time). The gel was treated with the stain solution (15 mL Milli-Q water, 1.4 mL 0.1 M NaOH, 100 μL concentrated 35% w/w ammonia, 250 μL of 20% silver nitrate) for 10 min in an orbital shaker (75 rpm). The solution was removed before washing the gel three times with distilled water (15 s each time). After all washes, a pre-warmed 40 °C developer solution (100 μL 3% citric acid, 25 μL 30% formaldehyde, 50 mL Milli-Q water) was added and incubated in darkness. When the bands were visible, the developer solution was removed, and the gel was washed with distilled water.

## Results

### Characteristic of OMVs Extracted From *S.* Typhi WT and Hypervesiculating Mutant Derivatives

**FIGURE 1:**
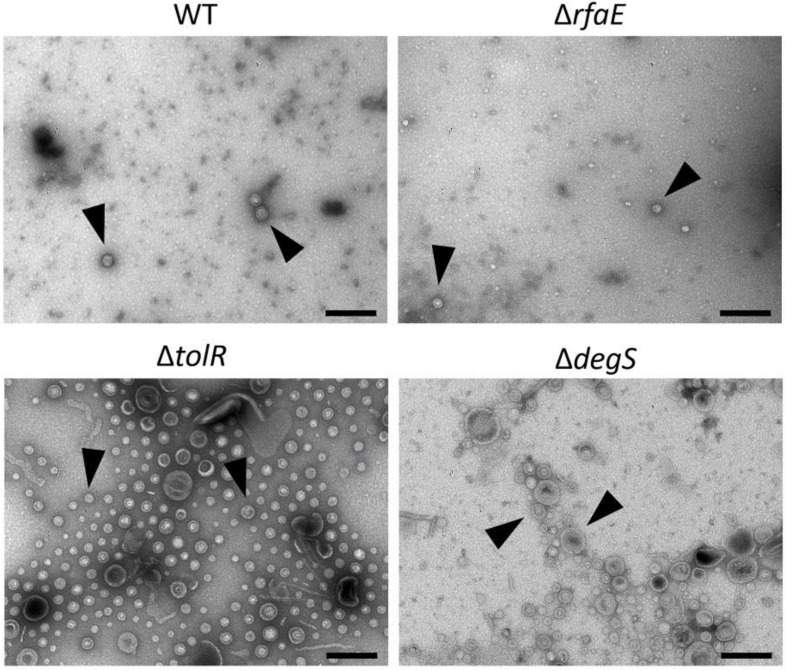
OMVs produced by *S.* Typhi WT, Δ*rfaE*, Δ*tolR*, and Δ*degS* mutants. Bacteria were grown in LB to OD_600_ = 1.0 before extracting OMVs. OMV extracts were observed by transmission electron microscopy (TEM). Arrowheads show OMVs. The black bar corresponds to 200 nm. In all cases, the horizontal field width and the magnification corresponded to 1.5 μm and 60,000×, respectively. In each case, a representative experiment is shown (*n* = at least 3).

To show some features of the OMVs under study, we characterize them by TEM, showing different morphologies and abundance (Figure 1). In addition, we determined their size (Table 1), showing that the OMVs from *S.* Typhi Δ*tolR* and Δ*degS* present a bigger size than OMVs from *S.* Typhi WT and Δ*rfaE*. Also, we determined the Zeta potential, where OMVs from *S.* Typhi Δ*degS* showed a more negative value. Previously, it has been reported that these OMVs present different protein content (Nevermann et al., 2019), which could contribute to such differences. All these results show the OMVs under study present distinct features.

**TABLE 1:** Some characteristics of OMVs used in this study.

Source of OMVs	OMV diameter (nm) Median (P50)^*a*^	OMV diameter (nm) P25	OMV diameter (nm) P75	Zeta potential (mV)^*b*^
*S.* Typhi WT	25	20	28	−13.50 ± 2.07
*S.* Typhi Δ*rfaE*	22	19	28	−12.35 ± 1.44
*S.* Typhi Δ*tolR*	41***	36	45	−11.30 ± 1.22
*S.* Typhi Δ*degS*	54***	43	65	−20.15 ± 0.89*

*n = at least 3.*

^a^Kruskal-Wallis, Dunn as a post hoc test, ***p < 0.001. The analysis refers to differences in the size among different OMVs, not only to the median.

^b^One-way ANOVA, Tukey as a post hoc test, *p < 0.05.

### OMVs Obtained From *S.* Typhi Mutants Increased the MIC of Polymyxin B in a Concentration-Dependent Manner

**FIGURE 2:**
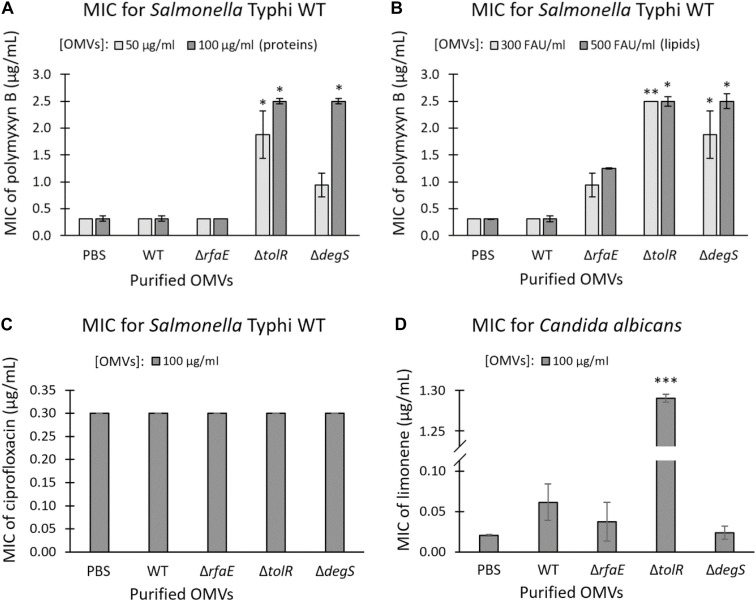
Purified OMVs from *S.* Typhi mutant derivatives contributed to the resistance of polymyxin B and limonene. MIC of polymyxin B for *S.* Typhi WT supplemented with OMVs extracted from *S.* Typhi WT, Δ*rfaE*, Δ*tolR*, or Δ*degS* **(A,B)**. OMV concentration was standardized as protein **(A)** or lipide content **(B)** and expressed as the final concentration. **(C)** MIC of ciprofloxacin for *S.* Typhi WT supplemented with OMVs extracted from *S.* Typhi WT, Δ*rfaE*, Δ*tolR*, or Δ*degS*. OMVs were standardized by protein content. **(D)** MIC of limonene for a clinical isolate of *Candida albicans* supplemented with OMVs extracted from *S.* Typhi WT, Δ*rfaE*, Δ*tolR*, or Δ*degS*. OMVs were standardized by protein content. In all cases, PBS was used as the negative control. FAU: Fluorescent arbitrary units obtained with the FM4-64 probe (Nevermann et al., 2019) as a measure of lipid concentration. (*n* = at least 4; bars represent the standard error; One-way ANOVA, Tukey as a *post hoc* test, ^∗^*p* < 0.05; ^∗∗^*p* < 0.01; ^∗∗∗^*p* < 0.001; compared with PBS).

OMVs contribute to the resistance against some antimicrobial compounds, including polymyxin in *Escherichia coli* and *Pseudomonas aeruginosa* (Grenier et al., 1995; Kobayashi et al., 2000; Manning and Kuehn, 2011; Roszkowiak et al., 2019). In this context, we determined the MIC of polymyxin B for *S.* Typhi WT in the presence OMVs extracted from *S.* Typhi WT, Δ*rfaE*, Δ*tolR*, or Δ*degS*. As shown in Figures 2A,B, the presence of OMVs from *S.* Typhi Δ*tolR* or Δ*degS* increased the MIC of polymyxin B for *S.* Typhi WT (~0.3125 μg/mL) 3–8 times. Nevertheless, OMVs extracted from *S.* Typhi WT or *S.* Typhi Δ*rfaE* did not significantly increase the MIC of polymyxin B. As a control, we tested whether purified OMVs could protect against ciprofloxacin, a quinolone that inhibits DNA gyrase and topoisomerase IV (Zhang et al., 2018), i.e., it is not a membrane-active antibiotic. As shown in Figure 2C, the presence of OMVs did not increase the MIC of ciprofloxacin for *S.* Typhi WT. To determine whether the OMVs can protect against other membrane-acting antimicrobials, we tested the limonene’s antifungal effect. Limonene is a monoterpene that induces membrane stress in *Candida albicans*, producing oxidative stress leading to DNA damage and apoptosis (Thakre et al., 2021). Figure 2D shows that only the presence of OMVs extracted from *S.* Typhi Δ*tolR* increased the MIC of limonene. The other OMV extracts showed no effect in this case. All these results show that OMVs from *S.* Typhi Δ*tolR* and Δ*degS* protect *S.* Typhi WT against polymyxin B, and OMVs *S.* Typhi Δ*tolR* protects *Candida albicans* against limonene.

**FIGURE 3:**
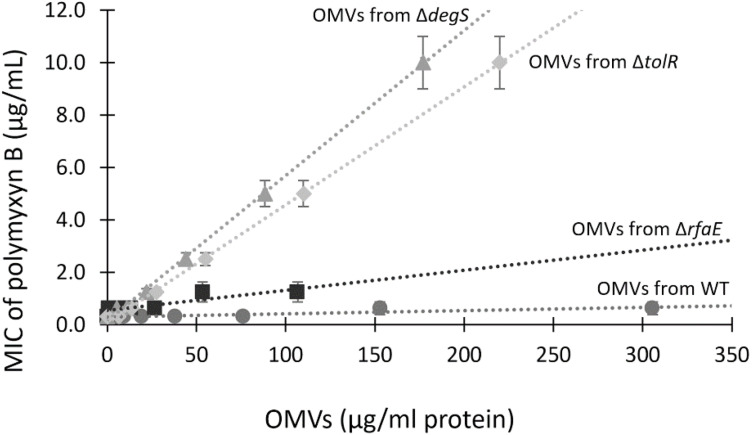
The MIC of polymyxin B depends on the concentration of OMVs. MIC of polymyxin B for *S.* Typhi WT was determined in the presence of different known concentrations of OMVs. Linear trend lines are represented by dotted lines (*n* = at least 4; bars represent the standard error).

To establish whether OMVs offer concentration-dependent protection against polymyxin B, we tested the MIC in the presence of increasing concentrations of OMVs. As shown in Figure 3, augmenting the concentration of OMVs from *S.* Typhi WT or *S.* Typhi Δ*rfaE* bearly increased the MIC of polymyxin B, even with the highest concentrations tested. By contrast, the presence of OMVs from *S.* Typhi Δ*tolR* or *S.* Typhi Δ*degS* showed a high protective effect, increasing almost linearly the protection under the range of concentration tested. The concentration-dependence protection against polymyxin B offered by OMVs (especially OMVs extracted from *S.* Typhi Δ*tolR* and Δ*degS*) is consistent with the role of OMVs capturing polymyxin B and decreasing its effective concentration.

### OMVs From *S.* Typhi Exert Their Protective Effect by Sequestering Polymyxin B

**FIGURE 4:**
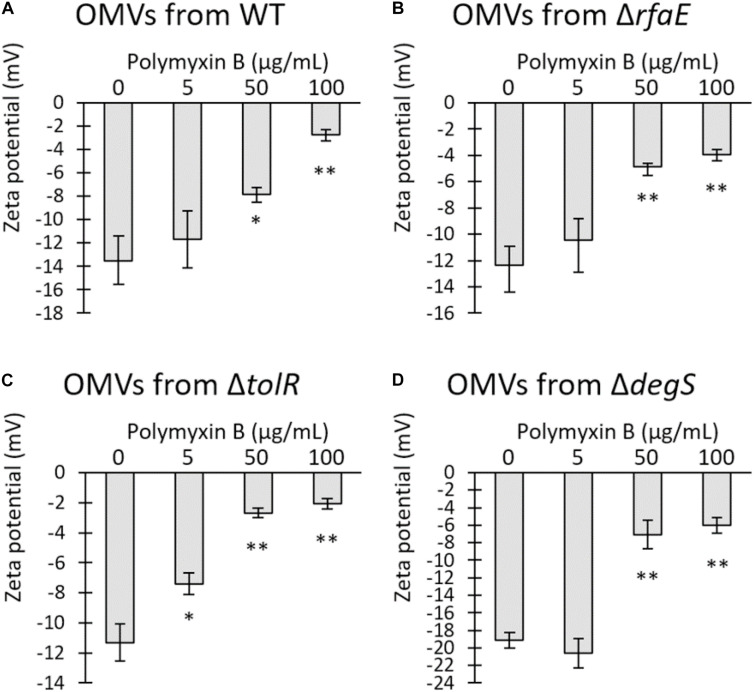
Polymyxin B interacts with OMVs from *S.* Typhi, depolarizing the Zeta potential of OMVs. Increasing concentrations of polymyxin B were mixed with 50 μg/mL OMVs obtained from *S.* Typhi WT **(A)**, Δ*rfaE* **(B)**, Δ*tolR* **(C)**, or Δ*degS* **(D)**. The mixture was incubated for 30 min at 37 °C and ultrafiltered to remove the unbound polymyxin B prior to determining the Zeta potential. (n = 3, bars represent standard error, One-way ANOVA, Tukey as a *post hoc* test, ^∗^*p* < 0.05; ^∗∗^*p* < 0.01; compared the control with no polymyxin B).

Previous works showed that OMVs from *Escherichia coli* and *Pseudomonas aeruginosa* sequester polymyxin B, decreasing the free concentration of the antibiotic (Kulkarni et al., 2015; Roszkowiak et al., 2019). The Zeta potential has been used as an indicator of interaction between bacterial membranes or OMVs with polymyxin B (Halder et al., 2015; Roszkowiak et al., 2019). Usually, OMVs present a negative Zeta potential, which can be depolarized by the interaction with cationic peptides such as polymyxin B. Thus, we mixed 50 μg/mL OMVs obtained from *S.* Typhi WT or mutant derivatives with polymyxin B. The mixture was incubated for 30 min at 37°C, and then ultrafiltered to remove any unbound polymyxin B prior to determining the Zeta potential of OMVs. As shown in Figure 4, the presence of polymyxin B tended to neutralize the Zeta potential of OMVs in a concentration-dependent manner, showing that the antibiotic remained retained by the vesicles. Interestingly, only OMVs from *S.* Typhi Δ*tolR* showed a significant depolarization with the lowest concentration tested. In all other cases, significant depolarization was achieved only with 50 μg/mL polymyxin B. This result suggests that OMVs from *S.* Typhi Δ*tolR* have a higher affinity for polymyxin B, plausibly removing more efficiently the polymyxin B from the medium and lowering the effective concentration.

To estimate the relative amount of polymyxin B sequestered by OMVs, we mixed 50 μg/mL OMVs extracted by *S.* Typhi WT or mutant derivatives with 100 μg/mL polymyxin B. We incubated for 30 min at 37 °C with gentle shaking before discarding the OMVs by ultrafiltration. To determine the relative amount of the unbound polymyxin B, the ultrafiltered fraction was diluted 10-fold in LB and then serially diluted in LB prior to being seeded with *S.* Typhi WT. The inhibition of bacterial growth was used as an indicator for the presence of polymyxin B. Table 2 shows that all the OMVs decreased the effective concentration of polymyxin B compared to the control. Furthermore, OMVs showed differential polymyxin-B sequestration abilities, in decreasing order: OMVs from Δ*tolR*, Δ*degS*, Δ*rfaE*, and WT. These results agree with those showing the protective effect of OMVs (Figure 3) and the Zeta potential determination (Figure 4). We obtained similar results when we determined the sequestration of polymyxin B by *S.* Typhi WT and mutant derivatives instead of OMVs (Supplementary Figure 1).

**TABLE 2:** Bioassay to determine the relative amount of polymyxin B in a solution previously incubated with OMVs extracted from *S.* Typhi WT or derivatives.

Source of OMVs	Last dilution that inhibited the *S.* Typhi WT growth (% v/v)^*a*^
No OMVs (control)	0.3
*S.* Typhi WT	1.3
*S.* Typhi Δ*rfaE*	2.5
*S.* Typhi Δ*tolR*	10.0
*S.* Typhi Δ*degS*	5.0

*^a^50 μg/mL OMVs were mixed with 100 μg/mL polymyxin B, incubated 30 min at 37°C with gentle shaking. OMVs were removed by ultrafiltration. Then, the filtrated was serially diluted in LB before being seeded with S. Typhi WT as a bioindicator for the presence of polymyxin B. n = 3 (this is a representative experiment).*

Thus, all these results together show that OMVs from *S.* Typhi exert their protective effect against polymyxin B by sequestering the antibiotic. Moreover, the OMVs from *S.* Typhi WT and mutant derivative are not equivalent, showing that mutations affecting different processes associated with OMV biogenesis generate OMVs with diverse properties.

### *S.* Typhi Δ*tolR* and Δ*degS* Can Functionally Transfer Their Polymyxin Resistance to Polymyxin-Susceptible Bacteria

**FIGURE 5:**
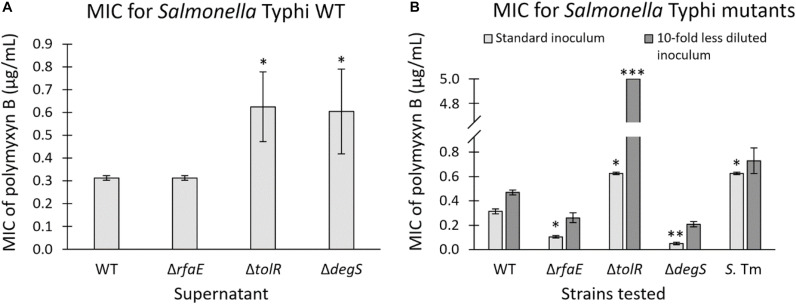
**(A)** MIC of polymyxin B for *S.* Typhi WT in the presence of supernatant (1:20 v/v) of *S.* Typhi WT, Δ*rfaE*, Δ*tolR*, or Δ*degS*. Supernatants were obtained from bacteria grown in LB at 37°C with shaking (OD_600_: 1.0–1.3). (*n* = at least 4). **(B)** MIC of polymyxin B of strains *S.* Typhi WT, Δ*rfaE*, Δ*tolR* or Δ*degS*, and *S.* Typhimurium LT2 *ompD*::Mu*d*J (*S.* Tm). We used two bacterial concentrations to seed the wells for the MIC determination (*n* = at least 4). In all cases, bars represent the standard error; One-way ANOVA, Tukey as a *post hoc* test, ^∗^*p* < 0.05; ^∗∗^*p* < 0.01; ^∗∗∗^*p* < 0.001; compared with *S.* Typhi WT under the same condition.

Our results show that purified OMVs from *S.* Typhi Δ*tolR* and Δ*degS* exert the highest protective effect against polymyxin B. In this context, we wondered whether the amount of OMVs present in the supernatant of these mutants is sufficient to protect against polymyxin B. Thus, we determined the MIC of polymyxin B for *S.* Typhi WT in the presence of supernatant from *S.* Typhi WT, Δ*rfaE*, Δ*tolR*, or Δ*degS*. We added 10 μL of the corresponding filtered supernatant to a final volume of 200 μL to determine the MIC of polymyxin B. As shown in Figure 5A, the supernatant of *S.* Typhi Δ*tolR* and Δ*degS*, diluted 20 times, was sufficient to increase two-fold the MIC of polymyxin B. By contrast, the supernatant obtained from *S.* Typhi WT or Δ*rfaE* showed no noticeable effects. Since OMVs are produced and released to the supernatant fraction during the normal bacterial growth (Kulp and Kuehn, 2010), we hypothesized that the *S.* Typhi Δ*tolR* and *S.* Δ*degS* mutants might protect surrounding bacteria against polymyxin B by producing and releasing protective OMVs. However, to exert a protective effect, OMV-producing bacteria should present an increased MIC to polymyxin B in order to reproduce and release sufficient OMVs to achieve the OMV-mediated protection. Thus, we determined the MIC of polymyxin B for the strains under study. As shown in Figure 5B (light gray bars), *S.* Typhi Δ*rfaE* and Δ*degS* exhibited a diminished MIC of polymyxin B (30% and 20% the MIC of the *S.* Typhi WT, respectively) (see a summary in Table 3). By contrast, *S.* Typhi Δ*tolR* showed a twofold increased MIC of polymyxin B. When we determined the MIC of polymyxin B but using 10-fold less diluted bacteria (dark gray bars), we found an increased resistance in all cases. Nevertheless, we observed a higher increase with the *S.* Typhi Δ*tolR* strain, which exhibited 10.7 times the MIC of the WT under the same conditions. Since polymyxin B can remain attached to biological membranes exerting a detergent-like effect (Velkov et al., 2013; Roszkowiak et al., 2019), it was expected that the MIC of polymyxin B would increase with a higher bacterial concentration. However, we postulate that the more notorious MIC rise showed by *S.* Typhi Δ*tolR*, compared with the WT, can also be explained by protective OMVs in the bacterial inoculum.

**TABLE 3:** MIC of polymyxin B for strains used in this study.

Strain	MIC of polymyxin B (μg/mL) ± SE
*S.* Typhi WT	0.31 ± 0.03
*S.* Typhi Δ*rfaE*	0.10 ± 0.05
*S.* Typhi Δ*tolR*	0.63 ± 0.08
*S.* Typhi Δ*degS*	0.05 ± 0.03
*S.* Typhimurium *ompD*::Mu*d*J	0.63 ± 0.05
*S.* Typhimurium *ompD*::Mu*d*J colonies recovered after de “one for all assay” (representative data of one colony)	0.63 ± 0.07

*SE, Standard error; n = at least 4.*

**FIGURE 6:**
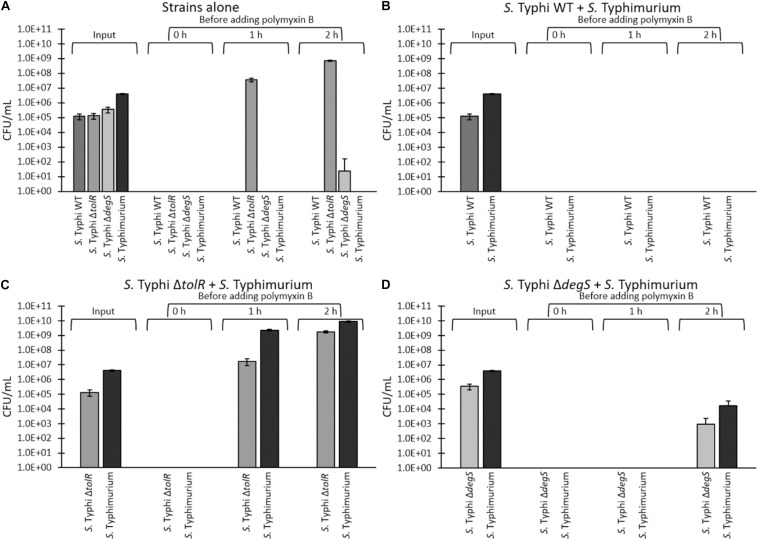
“One for all” assay of a challenge with 2.5 μg/mL polymyxin B. *S.* Typhi WT, Δ*tolR*, Δ*degS*, and *S*. Typhimurium *ompD*::Mu*d-*J (Lac^+^) were washed and diluted in LB to remove OMVs, and incubated in LB at 37°C with shaking for 0, 1, or 2 h at 37°C to allow OMVs accumulation, before adding polymyxin B (final concentration of 2.5 μg/mL, corresponding to 8 times the MIC of *S.* Typhi WT). Bacteria were incubated with polymyxin B overnight at 37°C with shaking prior to determining the CFU/mL (output). **(A)** Strains were challenged separately. **(B)** *S.* Typhi WT and *S.* Typhimurium *ompD*::Mu*d-*J were mixed (1:10) before the challenge. **(C)** *S.* Typhi Δ*tolR* and *S.* Typhimurium *ompD*::Mu*d-*J were mixed (1:10) before the challenge. **(D)** *S.* Typhi Δ*degS* and *S.* Typhimurium *ompD*::Mu*d-*J were mixed (1:10) before the challenge. To determine the CFU/mL, bacteria were plated onto LB supplemented with X-gal (white colonies: *S.* Typhi WT or derivatives, blue colonies: *S.* Typhimurium *ompD*::Mu*d*J). Alternatively, the serovar was corroborated by a PCR for the SPI-18 as previously described (Fuentes et al., 2008). (In all cases, *n* = at least 4).

To test the resistance of *S.* Typhi and the most potent hypervesiculating strains (*S.* Typhi Δ*tolR* and Δ*degS*) with an alternative procedure, we performed a challenge with a high polymyxin B concentration (2.5 μg/mL, corresponding to 8 times the MIC of the WT). To that aim, the strains (OD_600_ = ∼1.2) were washed three times and diluted in fresh LB to remove all OMVs previously accumulated in the supernatant (input). Then, bacteria were resuspended directly in LB supplemented with 2.5 μg/mL polymyxin B (time 0 h) or incubated in LB alone for 1 or 2 h at 37 °C with shaking to allow OMVs accumulation, before adding 2.5 μg/mL polymyxin. Bacteria were then incubated overnight at 37 °C with shaking prior to determining the final CFU/mL (output). As shown in Figure 6A, we were unable to recover colonies when we added polymyxin B immediately after washing (0 h), plausibly due to the absence of OMVs exerting a protective effect. However, when bacteria were incubated in LB for 1 or 2 h before adding polymyxin B, we could recover *S.* Typhi Δ*tolR.* We also recovered *S.* Typhi Δ*degS* but only after 2 h of incubation in LB before the polymyxin-B challenge. This result could be explained by accumulating protective OMVs during the incubation in LB prior to adding polymyxin B. The differences between *S.* Typhi Δ*tolR* and Δ*degS* strains can be attributed to their MIC of polymyxin B. By contrast, we could not recover *S.* Typhi WT after the challenge with polymyxin B, even after 2 h of incubation in LB.

If the growth of *S.* Typhi Δ*tolR* and Δ*degS* after the challenge with polymyxin B involves OMVs, this resistance should be functionally and transiently transferred to susceptible bacteria since OMVs are diffusible elements. Thus, we tested whether mutants can protect a polymyxin-susceptible strain (*S.* Typhimurium *ompD*::Mu*d*-J, Lac^+^) in an assay that we denominated “one for all.” *S.* Typhimurium *ompD*::Mu*d*-J has a MIC of polymyxin B corresponding to around twice the MIC exhibited by *S.* Typhi WT (∼0.625 μg/mL, Figure 5). In addition, we could not recover colonies of this strain in the challenge with 2.5 μg/mL, even after 2 h of incubation in LB before adding the antibiotic (Figure 6A), demonstrating its susceptibility to polymyxin B under the tested conditions. To perform the protection assay, *S.* Typhi WT, Δ*tolR*, or Δ*degS* were cultured, washed and diluted as described above, and mixed with *S.* Typhimurium *ompD*::Mu*d*-J (also washed and diluted) before the challenge with 2.5 μg/mL polymyxin B. The output was determined by plating onto LB with X-gal (white colonies: *S.* Typhi WT or mutant derivatives, blue colonies: *S.* Typhimurium *ompD*::Mu*d-*J). As shown in Figure 6B, *S.* Typhi WT could not protect the reporter strain even after 2 h of incubation in LB before adding polymyxin B and vice-versa.

In the *S.* Typhi Δ*tolR* + *S.* Typhimurium *ompD*::Mu*d*-J mixture, we observed that *S.* Typhi Δ*tolR* could resist the polymyxin challenge after 1 o 2 h of preincubation in LB. Accordingly, only the presence of *S.* Typhi Δ*tolR* efficiently protected the susceptible reporter strain since no colonies were seen with no preincubation (0 h) (Figure 6C). Consistent with a higher accumulation of protective OMVs, the mixture incubated for 2 h before the challenge with polymyxin B showed the highest protection for *S.* Typhi Δ*tolR* and *S.* Typhimurium *ompD*::Mu*d*-J.

Finally, the mixture of *S.* Typhi Δ*degS* + *S.* Typhimurium *ompD*::Mu*d*-J showed colonies of both bacteria only after 2 h of preincubation in LB (Figure 6D). Again, we attribute this result to the accumulation of protective OMVs. Despite the high degree of protection provided by OMVs from *S.* Typhi Δ*degS*, the lower protective effect of such mutant is consistent with its lower MIC of polymyxin B, compared with *S.* Typhi Δ*tolR*.

*S.* Typhimurium *ompD*::Mu*d*-J recovered after the challenge showed an unaffected MIC of polymyxin (∼0.625 μg/mL), showing that the protection received by *S.* Typhi Δ*tolR* or Δ*degS* is transient and could not be attributed to genetic changes (Table 3). Besides, it is important to state that the obtained results cannot be attributed to a faster-growing phenotype of *S.* Typhi Δ*tolR* or *S.* Typhi Δ*degS* (Table 4).

**TABLE 4:** Growth rate (μ) and duplication time (t_*d*_) of *S.* Typhi strains used in this study.

Strain	μ (h^–1^) ± SE^*a*^	t_*d*_ (min) ± SE^*a*^	*p*^*b*^
*S.* Typhi WT	1.532 ± 0.052	27.246 ± 0.967	–
*S.* Typhi Δ*rfaE*	1.539 ± 0.049	27.090 ± 0.846	ns
*S.* Typhi Δ*tolR*	1.480 ± 0.085	28.393 ± 1.765	ns
*S.* Typhi Δ*degS*	1.432 ± 0.067	29.235 ± 1.437	ns

*Strains were cultured in LB at 37°C with shaking. Bacterial growth was assessed by measuring OD_600_ over time.*

*^a^n = 4 (with 8 technical replicates). SE, standard error.*

*^b^One-way ANOVA, with Tukey as post hoc test. ns = non-significative compared with S. Typhi WT.*

**FIGURE 7:**
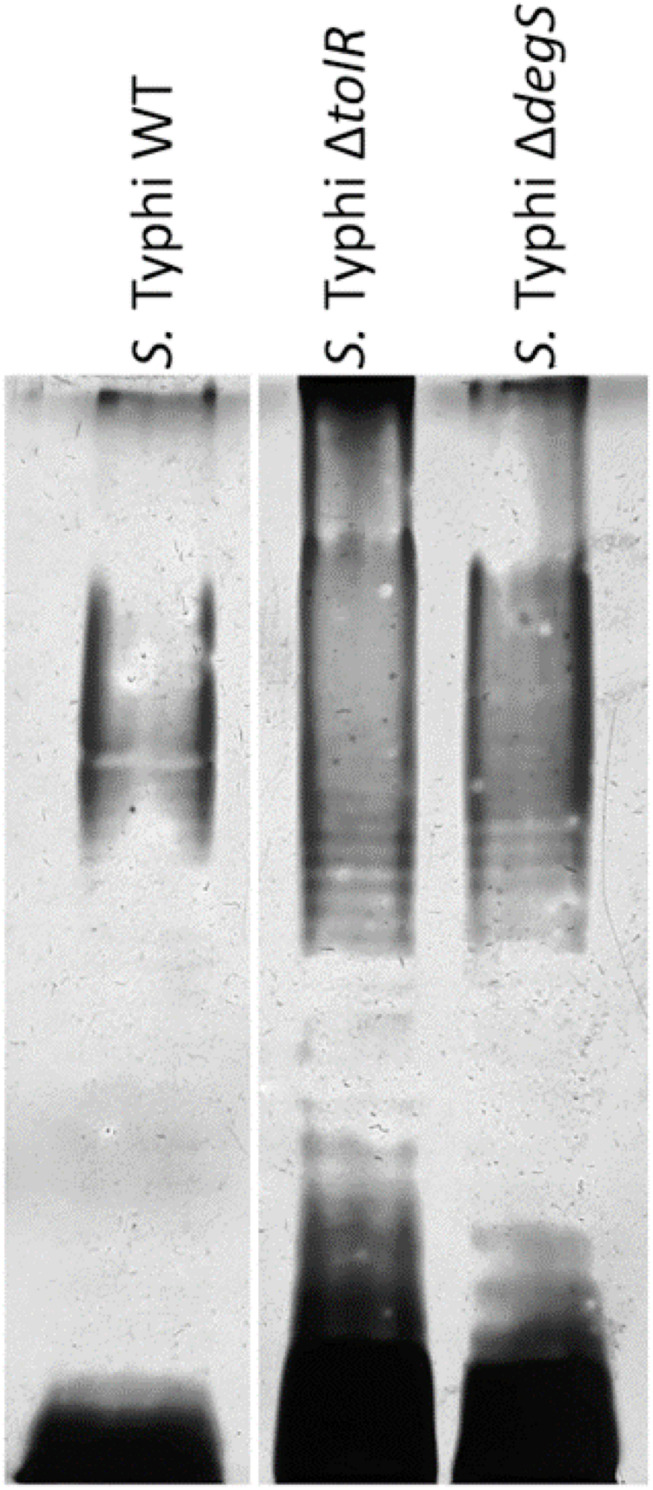
LPS of OMVs extracted from *S.* Typhi WT, Δ*tolR*, and Δ*degS*. OMVs were extracted as described, and the LPS profile was resolved as described in section “Materials and Methods.” In each lane, 40 μg/mL (proteins) of OMVs were loaded.

At this point, OMVs extracted from *S.* Typhi Δ*tolR* and Δ*degS* showed the most noticeable protective effect against polymyxin B. This result suggests that their compositions are different from the OMVs produced by the WT. Previously, it has been reported that the protein cargo of OMVs from *S.* Typhi WT, Δ*tolR*, and Δ*degS* are different among them (Nevermann et al., 2019). Since LPS is crucial regarding polymyxin interaction, we assessed the LPS profile in these OMVs to complement this information. As shown in Figure 7 and Supplementary Figure 2, the LPS profile of OMVs extracted from *S.* Typhi Δ*tolR* and Δ*degS* present a distinct pattern. These differences could also be contributing to the protective effect of these OMVs against polymyxin B.

All these results show that a susceptible strain efficiently can grow in the presence of a high amount of polymyxin B when is cocultured with a hypervesiculating strain producing protective OMVs. We observed this protective effect even with *S.* Typhi Δ*degS*, which presents a very low MIC of polymyxin B when this mutant encounters conditions that allow OMVs accumulation. This protective effect is more potent with a strain exhibiting a higher MIC of polymyxin B, as shown with *S.* Typhi Δ*tolR*.

## Discussion

In this work, we found that the OMVs from *S.* Typhi WT and mutant derivatives exert a protective effect against polymyxin B, albeit the OMVs from *S.* Typhi Δ*tolR* and Δ*degS* were much more protective. Furthermore, we found that *S.* Typhi Δ*tolR* (and at a lesser degree, *S.* Typhi Δ*degS*) can functionally transfer the polymyxin-resistance to susceptible bacteria, plausibly via OMVs. To our knowledge, this is the first report exploring OMVs from *S.* Typhi as protective agents against antimicrobial agents. Furthermore, this is the first study showing that OMVs obtained by different genetic backgrounds exhibit, in turn, different protective effects.

We found that the presence of purified OMVs exerted a protective effect against polymyxin B and limonene but not against ciprofloxacin. Both polymyxin B and limonene exert their antimicrobial effect by interacting with biological membranes, while ciprofloxacin targets gyrase and topoisomerase IV (Velkov et al., 2013; Zhang et al., 2018; Thakre et al., 2021). OMVs from *E. coli* can protect against membrane-active antibiotics, i.e., polymyxin B, colistin (polymyxin E) and melittin, but no against antibiotics with other targets, such as ciprofloxacin, streptomycin and trimethoprim (Manning and Kuehn, 2011; Kulkarni et al., 2015). Although polymyxin B and limonene do not share structural similarities, their modes of action require the interaction with biological membranes, suggesting that the protection exerted by *S.* Typhi OMVs is based on an unspecific mechanism involving membranes. In this sense, this work showed that OMVs from *S.* Typhi and mutant derivatives sequester polymyxin B and remove it from the solution. The fact that OMVs increased the MIC of polymyxin B in a concentration-dependent manner supports this assertion. Furthermore, in the present manuscript, the sequestration of polymyxin B by OMVs was shown by measuring the Zeta potential of OMVs exposed to polymyxin B and determining the polymyxin B activity that remained after coincubating with OMVs. A similar strategy was previously used to demonstrate the sequestration of polymyxin B by *Pseudomonas aeruginosa* OMVs (Roszkowiak et al., 2019), and this same mechanism was also shown in *E. coli* (Kulkarni et al., 2015). The data that we obtained with the Zeta potential agrees with the bioassay designed to assess the polymyxin B removed by OMVs, where we found that all OMVs tested showed the ability to decrease the amount of effective polymyxin B. According to our results and previous works showing the sequestration of polymyxin B by OMVs from other bacteria (Kulkarni et al., 2015; Roszkowiak et al., 2019), the most straightforward explanation is that OMVs from *S.* Typhi sequester polymyxin B. However, we cannot rule out that other mechanisms were also acting. Kulkarni et al. (2015) showed that OMVs from *Escherichia coli* sequester both colistin and melittin. Furthermore, they demonstrated that the OMVs from *Escherichia coli* also degrade melittin, but not colistin (Kulkarni et al., 2015). Thus, determining whether OMVs from *S.* Typhi can degrade polymyxin B, as an additional mechanism, remains to be elucidated.

A previous study showed that OMVs from *S.* Typhi WT, Δ*rfaE*, Δ*tolR*, and Δ*degS* present different features, such as size and protein content (Nevermann et al., 2019). Besides, *rfaE*, *tolR*, and *degS* are genetic determinants of three different processes involved in OMV biogenesis (Schwechheimer and Kuehn, 2015; Nevermann et al., 2019). It has been shown that mutations affecting different OMV biogenesis processes produce OMVs with different cargo (Altindis et al., 2014; Bonnington and Kuehn, 2014; Murphy et al., 2014; Schwechheimer and Kuehn, 2015). For these reasons, it was postulated that OMVs from *S.* Typhi WT, Δ*rfaE*, Δ*tolR*, and Δ*degS* should present different properties regarding biological functions (Nevermann et al., 2019). As stated above, polymyxin B, a cationic amphipathic peptide, can interact with negatively charged LPS, as well as with proteins, to get inserted into the outer membrane, interacting with the lipid A (Daugelavicius et al., 2000; Falagas and Kasiakou, 2006; Velkov et al., 2013; van der Meijden and Robinson, 2015; Trimble et al., 2016). Since OMVs are discharged from the outer membrane, mutations affecting the bacterial envelope also affect the OMV content, including both LPS and proteins (Kim et al., 2009; Liu et al., 2016a), potentially affecting the polymyxin—OMVs interaction or affinity. In the present manuscript, we found apparent differences among mutants, where OMVs can be sorted in decreasing protective effect as OMVs from *S.* Typhi Δ*tolR*, Δ*degS*, Δ*rfaE*, and WT. Why OMVs from *S.* Typhi Δ*tolR* showed the most potent protective effect against polymyxin B could be attributed to their higher affinity by polymyxin B as the Zeta potential measurement suggests and the sequestration bioassay showed.

Previously published works support the high affinity showed by OMVs from *S.* Typhi Δ*tolR* shown in the present study. The *tolR* gene encodes an inner membrane protein of the trans-envelope Tol-Pal complex (Sturgis, 2001). In *E. coli and S.* Typhimurium, TolR participates in maintaining the envelope structure and retrograde phospholipid transport (Muller et al., 1993; Masilamani et al., 2018; Boags et al., 2019). Furthermore, *E. coli* mutants in genes encoding Tol-Pal components showed defective O-antigen polymerization (Vinés et al., 2005), while *Pseudomonas aeruginosa* defective in a Tol-Pal component (TolA) showed membranes with high affinity for cationic compounds, including polymyxin B, due to changes in the LPS (Rivera et al., 1988). Furthermore, the hypervesiculating *Shigella flexneri* Δ*tolR* produces OMVs with alterations in the LPS O-chain (Pastor et al., 2018). Thus, we propose that OMVs from *S.* Typhi Δ*tolR* present a higher affinity for polymyxin B than the other OMVs tested due to changes in their membrane profile. On the other hand, we observed an increased MIC of polymyxin B for *S.* Typhi Δ*tolR* than the WT. Nevertheless, when the *S.* Typhi Δ*tolR* was washed, it was necessary, at least, to incubate bacteria for 1 h to obtain CFU after the challenge with 2.5 μg/mL polymyxin B, or to protect susceptible bacteria in a coculture. From these results, we inferred that the increased MIC of polymyxin B exhibited by *S.* Typhi Δ*tolR* can be attributed to OMVs. Nevertheless, other mechanisms can also be contributing to this phenotype. According to the Zeta potential experiments and sequestering of polymyxin B, OMVs from *S.* Typhi Δ*tolR* have the highest affinity by polymyxin B. In this sense, the bioassay of the polymyxin B activity after incubation with bacteria strongly suggests that the *S.* Typhi Δ*tolR* envelope has increased affinity for polymyxin B than the WT (Supplementary Figure 1). In this case, a membrane with a high affinity for polymyxin B might also increase antibiotic resistance via OMV production. *S.* Typhi Δ*tolR* presents one of the most hypervesiculating phenotypes in *S.* Typhi, showing a high release of OMVs when grown under standard conditions (LB, 37°C with shaking), without the need for additional stimuli (Nevermann et al., 2019). Thus, the polymyxin B bound to bacterial membranes might be rapidly discharged from the cells by the hyperproduction of OMVs. Supporting this point, it has been described a toluene elimination system in *Pseudomonas putida*, where the toluene adhered to the outer membrane is rapidly eliminated by shedding OMVs, rendering this strain resistant to such compound (Kobayashi et al., 2000). The role of OMVs to eliminate toxic compounds from the bacterial envelope has already been reviewed (Schwechheimer and Kuehn, 2015).

We also found that OMVs from *S.* Typhi Δ*degS* protect bacteria against polymyxin B. DegS regulates σ^*E*^ activation under membrane stress (Alba et al., 2001). In *Salmonella* Typhimurium, σ^*E*^ is required under oxidative stress, envelope stress, and the presence of antimicrobial peptides (Testerman et al., 2002; Palmer and Slauch, 2020). Crosstalk between outer membrane protein and LPS biogenesis with the activation of σ^*E*^ has been reported (Kim, 2015), suggesting a different composition in the lipidic components in OMVs from *S.* Typhi Δ*degS*, as found in Figure 7 and Supplementary Figure 2. The most negative Zeta potential in these OMVs could support this hypothesis. On the other hand, σ^*E*^ is necessary for resistance to cationic peptides in *S.* Typhimurium (Crouch et al., 2005). The lack of DegS may be impairing the σ^*E*^ activation, explaining the low MIC exhibited by the *S.* Typhi Δ*degS* mutant. In addition, although the *S.* Typhi Δ*degS* envelope seems to present a similar affinity for polymyxin B than *S.* Typhi Δ*tolR*, as inferred by Supplementary Figure 1, the OMV release by *S.* Typhi Δ*tolR* is 1,000 times more than the OMV release by *S.* Typhi Δ*degS* (measured as protein content). Thus, an envelope with increased affinity for polymyxin B but insufficient OMVs production could be considered detrimental regarding polymyxin resistance.

OMVs from *S.* Typhi WT and Δ*rfaE* showed low protection levels against polymyxin B. Nevertheless, the *S.* Typhi Δ*rfaE* strain exhibited a much lower MIC of polymyxin B than the WT strain. In *Salmonella* Typhimurium, it has been reported that Δ*rfaE* and other mutants involved in the LPS synthesis present an increased membrane permeability, decreasing the resistance to diverse antimicrobial compounds, including polymyxin B (Vaara, 1993; Acuna et al., 2016). In particular, *rfaP* (*waaP*), whose product is responsible for phosphorylation of the first heptose residue of the LPS inner core region, is necessary for polymyxin resistance in *E. coli*. The authors concluded that the absence of phosphoryl modifications in the LPS core region leads to an increased polymyxin susceptibility, despite the more depolarized membrane (Yethon et al., 2000). *Salmonella* Typhimurium Δ*rfaE* mutants synthesize heptose-deficient LPS (Jin et al., 2001) (i.e., no phosphorylation by WaaP would be possible), providing a possible explanation of the phenotype found with *S.* Typhi Δ*rfaE*.

Polymyxin mode of action requires two main kinds of interactions to get inserted into the bacterial outer membrane. (1) Electrostatic interaction between the cationic moiety of polymyxin and negatively charged LPS, and (2) hydrophobic interaction between the aliphatic acyl tail of polymyxin and hydrophobic segments of the membrane, including lipid A (Velkov et al., 2010; Trimble et al., 2016). The evidence also suggests polymyxin interaction with outer membrane proteins (van der Meijden and Robinson, 2015). In this sense, OMV from *S.* Typhi Δ*tolR* shows the highest sequestering ability (Figure 4 and Table 2), albeit their Z potential is similar to that observed with OMVs from the WT (Table 1). This result suggests that the hydrophobic interaction might be most important concerning the increased protection ability of OMVs from *S.* Typhi Δ*tolR*. On the other hand, OMVs from Δ*degS* could be exerting their protective effect by increasing the electrostatic interactions with polymyxin B, as their more negative Z potential suggests (Table 1). Nevertheless, since OMVs are complex supramacromolecular entities, both the hydrophobicity and the negative charge can be achieved by a complex interaction of protein and lipid cargo. Accordingly, OMVs from *S*. Typhi Δ*tolR* and Δ*degS* showed a pattern of protein cargo that is different from OMVs extracted from the WT (Nevermann et al., 2019), where preliminary proteomic analyses show that OMVs from *S.* Typhi Δ*tolR* and Δ*degS* have approximately 180 and 500 proteins, respectively, absent from OMVs from the WT (unpublished results). Furthermore, OMVs from *S.* Typhi WT and mutant derivatives show different LPS profiles (Figure 7 and Supplementary Figure 2), strongly suggesting that increased affinity for polymyxin B is multifactorial.

At present, the role of OMVs as protective agents against polymyxin, or other antimicrobial compounds, has been assessed by extracting OMVs and adding them to axenic reporter cultures to determine the degree of protection (Manning and Kuehn, 2011; Kulkarni et al., 2015; Roszkowiak et al., 2019). However, studies showing the participation of OMVs in more physiological conditions are less common. In this study, we tested whether the hypervesiculating strains could protect a susceptible strain from a challenge with a high amount of polymyxin B in a coculture. We found that, when the strains were washed to remove OMVs, no colonies were observed after the challenge. Nevertheless, when the culture was incubated for 1 h to allow the bacterial growth and OMV accumulation, we found that *S.* Typhi Δ*tolR* could resist the challenge with polymyxin B. Longer incubation times allowed even the *S.* Typhi Δ*degS* growth. We inferred that the survival of *S.* Typhi Δ*tolR* and, at a lesser level, *S.* Typhi Δ*degS*, depends on the OMV accumulation. Consistently, both strains could transiently transfer their polymyxin B resistance to a susceptible reporter strain. Since the reporter strains did not show an increased MIC of polymyxin B after the challenge, we ruled out any genetic change. Altogether, these results argue for a functional and transient transfer of OMV-mediated polymyxin B resistance from *S.* Typhi Δ*tolR* and Δ*degS* to susceptible bacteria in more physiological conditions, i.e., in a coculture. The most potent protective effect shown by *S.* Typhi Δ*tolR* is consistent with the high protection level of its OMVs, the apparent higher affinity of its OMVs for polymyxin B, and the increased MIC of polymyxin B. It has been reported that mutations leading to changes in the bacterial envelope can increase the resistance to polymyxin B by decreasing the anionic charges (Olaitan et al., 2014; Li et al., 2019). Nevertheless, this kind of resistance could be considered “selfish” since it is not generally thought to be shared, except for the *mcr* genes (Olaitan et al., 2014). However, in this study, we showed that it is possible to transfer the polymyxin resistance via OMVs to the bacterial community without genetic exchange.

This work showed that mutants in genes related to OMVs biogenesis can release vesicles with improved abilities to protect bacteria against membrane-active agents such as polymyxin B. Since mutations affecting OMV biogenesis can involve the bacterial envelope (Kulp and Kuehn, 2010; Kulkarni and Jagannadham, 2014; Nevermann et al., 2019), it is possible to obtain mutant bacteria with increased resistance to membrane-acting agents that, in turn, produce protective OMVs with a high vesiculation rate (e.g., S. Typhi Δ*tolR*). Such mutants can functionally transfer the resistance to surrounding bacteria via OMVs, diminishing the effective concentration of the antimicrobial agent and potentially favoring the selection of spontaneous resistant strains in the environment. Finally, since OMVs can also protect against other agents such as antimicrobial peptides, which can be produced by the innate immune system (Urashima et al., 2017), the possible role of vesicles in bacterial pathogenesis as protective agents is progressively gaining attention.

## Data Availability Statement

The original contributions presented in the study are included in the article/Supplementary Material, further inquiries can be directed to the corresponding author/s.

## Author Contributions

PM: experiments, support, and discussion. AC: facilities, Zeta potential experiments, and discussion. EV: experiments and support. AS: TEM and support. JN: mutant construction and support. CO: manuscript edition. EA: facilities, supervision of Zeta potential experiments. FG: facilities, discussion, and manuscript edition. IC: facilities, discussion, and manuscript edition. JF: conception of the study, data curation, figures, facilities, funding acquisition, manuscript writing, and editing. All authors contributed to the article and approved the submitted version.

## Conflict of Interest

The authors declare that the research was conducted in the absence of any commercial or financial relationships that could be construed as a potential conflict of interest.
