# Supplementary material for: “One for All”: Functional Transfer of OMV-Mediated Polymyxin B Resistance From Salmonella enterica sv. Typhi ΔtolR and ΔdegS to Susceptible Bacteria
Source: Front Microbiol. 2021 May 5;12:672467. doi: 10.3389/fmicb.2021.672467 (PMC8131662; doi:10.3389/fmicb.2021.672467)
Supplement: Supplementary file 1 [file Data_Sheet_1.docx]

**“One for all”:** **Functional transfer of OMV-mediated polymyxin B resistance from *Salmonella* enterica sv. Typhi Δ*tolR* and Δ*degS* to susceptible bacteria**

Pedro Marchant,^a^ Alexander Carreño,^b^ Eduardo Vivanco,^a^ Andrés Silva,^a^ Jan Nevermann,^a^ Carolina Otero,^c^ Eyleen Araya,^d^ Fernando Gil,*^e,f^ Iván L. Calderón,*^g^ Juan A. Fuentes*^a^

^a^Laboratorio de Genética y Patogénesis Bacteriana, Departamento de Ciencias Biológicas, Facultad de Ciencias de la Vida, Universidad Andres Bello, Chile

^b^Center of Applied NanoSciences (CANS), Facultad de Ciencias Exactas, Universidad Andres Bello, Santiago, Chile.

^c^Escuela de Química y Farmacia, Facultad de Medicina, Universidad Andres Bello, Chile.

^d^Departamento de Ciencias Químicas, Facultad de Ciencias Exactas, Universidad Andres Bello, Chile.

^e^Microbiota-Host Interactions and Clostridia Research Group, Universidad Andres Bello, Chile.

^f^ANID-Millennium Science Initiative Program-Millennium Nucleus in the Biology of the Intestinal Microbiota, Santiago, Chile.

^g^Laboratorio de RNAs Bacterianos, Departamento de Ciencias Biológicas, Facultad de Ciencias de la Vida, Universidad Andres Bello, Chile.

*Corresponding authors:

Juan A. Fuentes ([jfuentes@unab.cl](mailto:jfuentes@unab.cl))

Iván L. Calderón ([lcalderon@unab.cl](mailto:lcalderon@unab.cl))

Fernando Gil ([fernandogil@unab.cl](mailto:fernandogil@unab.cl))

**Supplementary figures**


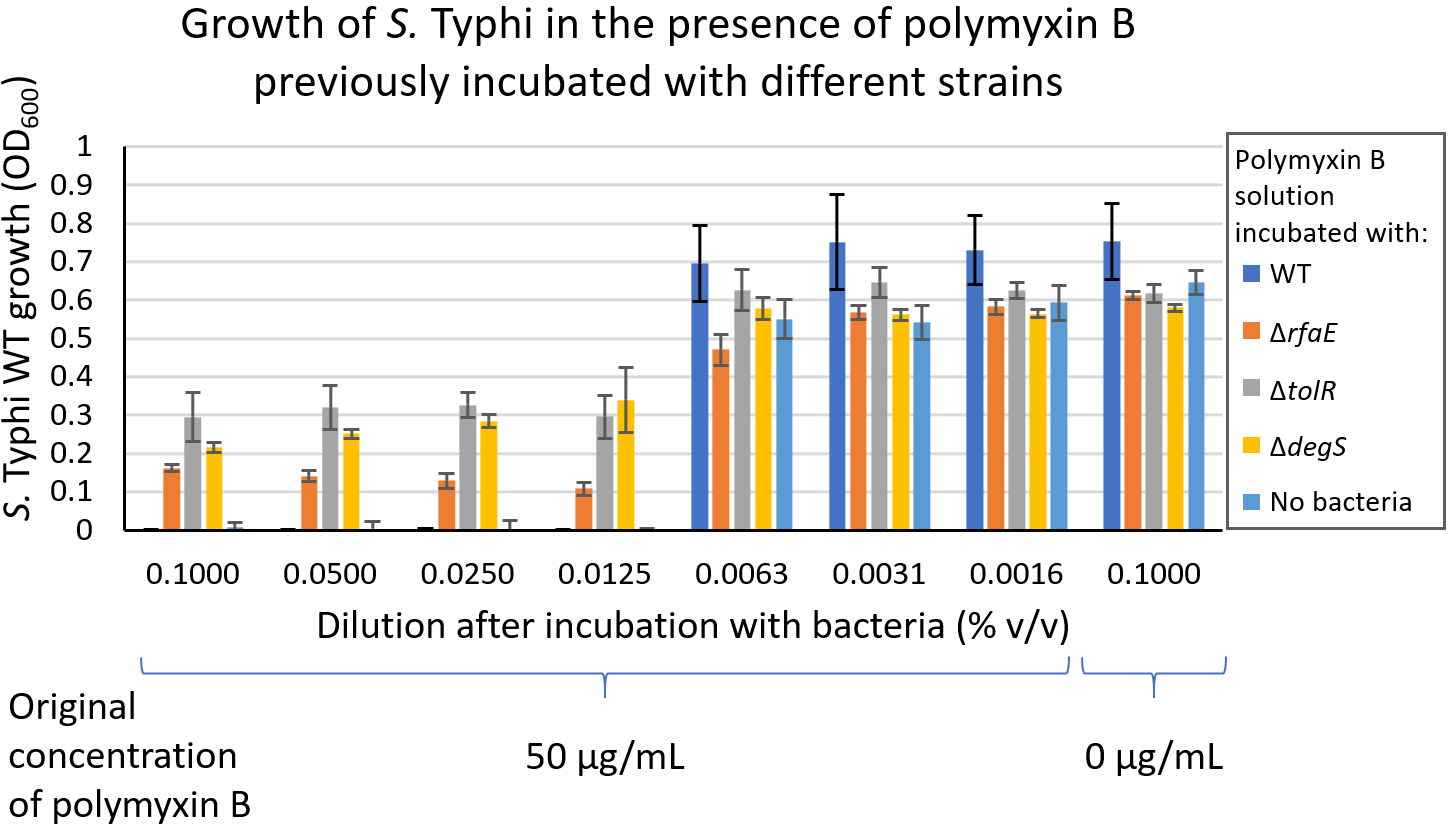


**Figure S1.** Bioassay to determine the relative amount of polymyxin B in a solution previously incubated with *S.* Typhi WT or derivatives. Bacteria (*S.* Typhi WT or mutant derivatives) were cultured overnight in LB at 37 °C with shaking. Approximately 10^7^ UFC/mL of each strain were washed three times with PBS to remove all OMVs, and resuspended in PBS. Bacteria were mixed with polymyxin B prepared in PBS (50 µg/mL or 0 µg/mL as control) and incubated at 37 °C with gentle shaking for 30 min. Bacteria were discarded by centrifugation (10 min at 5400 ×*g* at 4 °C), and the supernatant was ultrafiltered in Ultracel® 100 kDa ultrafiltration column (Amicon® Bioseparations) at 5400 ×*g* for 10 min to remove bacteria and any OMVs that could be produced during the incubation. The ultrafiltrated was serially diluted in LB, and the growth of *S.* Typhi WT was used to estimate the presence of polymyxin B. The X axis represents the dilution of the original stock of polymyxin B (50 µg/mL or 0 µg/mL as control). The dilution of 0.0063 % v/v corresponds to the MIC (0.315 µg/mL) for polymyxin B when no bacteria (control) were added to sequester polymyxin B. The OD_600_ is inversely proportional to the polymyxin B concentration in the ultrafiltrate. (n = 3).

**
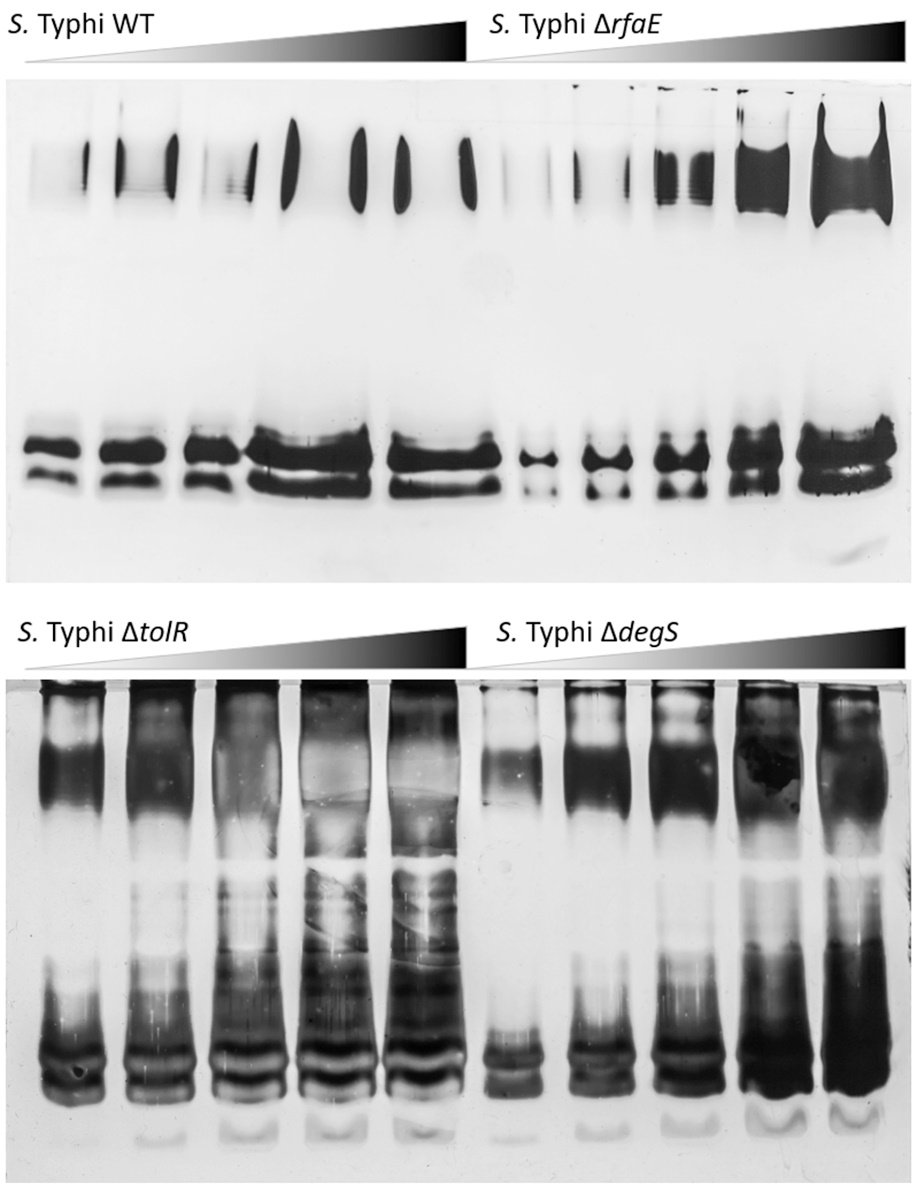
**

**Figure S2.** LPS of OMVs extracted from *S.* Typhi WT, Δ*rfaE*, Δ*tolR*, and Δ*degS*. OMVs were extracted as described, and the LPS profile was resolved as described in Materials and Methods. The amount of OMVs loaded for each strain was determined by the protein content (10 µg/mL, 20 µg/mL, 30 µg/mL, 40 µg/mL and 50 µg/mL).

**Supplementary Tables**

**Table S1.** MIC of limonene for strains used in this study

| **Strain** | **MIC of limonene (µg/mL) ± SE*** |
| --- | --- |
| *S.* Typhi WT | 0.042 ± 0.006 |
| *S.* Typhi Δ*rfaE* | 0.029 ± 0.006 |
| *S.* Typhi Δ*tolR* | 0.100 ± 0.000 |
| *S.* Typhi Δ*degS* | 0.367 ± 0.000 |

SE: Standard error; n = at least 4
